# Use of aids for smoking cessation and alcohol reduction: A population survey of adults in England

**DOI:** 10.1186/s12889-016-3862-7

**Published:** 2016-12-08

**Authors:** Emma Beard, Jamie Brown, Susan Michie, Eileen Kaner, Petra Meier, Robert West

**Affiliations:** 1 Research Department of Clinical, Educational and Health Psychology, University College London, London, UK; 2 Department of Epidemiology and Public Health, University College London, London, UK; 3 Institute of Health & Society, Newcastle University, Newcastle, UK; 4 ScHARR, The University of Sheffield, Sheffield, UK

**Keywords:** Smoking, Alcohol, High-risk drinking, Smoking Toolkit Study, Alcohol Toolkit Study, Treatment, Pharmacotherapy, Behavioural support

## Abstract

**Background:**

It is important for policy planning to chart the methods smokers and high-risk drinkers use to help them change their behaviour. This study assessed prevalence of use, and characteristics of users, of support for smoking cessation and alcohol reduction in England.

**Methods:**

Data were used from the Smoking and Alcohol Toolkit Studies, which involve monthly face-to-face computer-assisted interviews of adults aged 16+ in England. We included data collected between June 2014 and July 2015 on 1600 smokers who had made at least one quit attempt and 911 high-risk drinkers (defined as scores >8+ on the full AUDIT or 5+ on questions 1–3 of the AUDIT-C) who had made an attempt to cut down in the past 12 months. Participants provided information on their socio-demographic characteristics and use of aids during their most recent quit attempt including pharmacotherapy, face-to-face counselling, telephone support, self-help materials (digital and printed), and complementary medicine.

**Results:**

A total of 60.3% of smokers used aids in the past year, compared with just 14.9% of high-risk drinkers. Use of pharmacotherapy was high among smokers and very low among drinkers (56.0%_versus_1.2%). Use of other aids was low for both behaviours: face-to-face counselling (2.6%_versus_4.8%), self-help materials (1.4%_versus_4.1%) and complementary medicine (1.0%_versus_0.5%). Use of aids was more common among smokers aged 25–54 compared with 16–24 year olds (25–34,OR_adj_1.49,*p =* 0.012; 35–44,OR_adj_1.93,*p* < 0.001; 35–44,OR_adj_1.93,*p* < 0.001; 45–54,OR_adj_1.66,*p* = 0.008), with cigarette consumption >10 relative to <1 (10–20,OR_adj_2.47,*p* = 0.011; >20,OR_adj_4.23,*p* = 0.001), and less common among ethnic minorities (OR_adj_0.69,*p* = 0.026). For alcohol reduction, use of aids was higher among ethnic minority groups (OR_adj_2.41;*p* = 0.015), and those of social-grade D/E relative to AB (OR_adj_2.29,*p* = 0.012&OR_adj_3.13,*p* < 0.001).

**Conclusion:**

In England, the use of pharmacotherapy is prevalent for smoking cessation but not alcohol reduction. Other aids are used at a low rate, with face-to-face counselling being more common for alcohol reduction than smoking cessation.

**Electronic supplementary material:**

The online version of this article (doi:10.1186/s12889-016-3862-7) contains supplementary material, which is available to authorized users.

## Background

Smoking and alcohol consumption contribute to more than 7 million deaths per year worldwide and are associated with a wide range of chronic conditions [1–3]. National guidelines around the world recommend use of evidence-based aids for alcohol misuse and tobacco dependence to reduce this burden (e.g. England, America, Brazil, Uruguay and China) [4–7]. This includes a combination of behavioural support and pharmacotherapy [8, 9]. There are also a number of other aids available including self-help materials and complementary alternative medicines [10–13]. It is important to know what proportion of people make use of these aids in order to help develop policies aimed at maximising their impact. For example, if smokers are underusing Stop Smoking Services, which is one of the most cost-effective smoking cessation methods, Local Authorities responsible for the services may be encouraged to improve funding [8]. It is also important to understand the characteristics of users of aids to help target services effectively. This paper addresses those issues.

To our knowledge, no population survey has assessed the use of aids for alcohol reduction in England. A comprehensive evaluation of the use of smoking treatments was undertaken in 2008 [14]. However, given the changing climate of tobacco control since that time a different population profile may exist [15]. That study also did not report on aids such as acupuncture and hypnotherapy, digital interventions or self-help books. Thus this study attempted to assess self-reported use of aids among smokers and high-risk drinkers in England during 2014/2015. It also aimed to establish the socio-demographic and smoking or drinking characteristics profile of those using aids.

There are a number of reasons to believe that use of treatments among smokers may have changed since 2008 in England. Nicotine replacement therapy (NRT) has been licensed for both smoking cessation and smoking reduction, a wider range of pharmacological products are available on the market (e.g. electronic cigarettes), and control of stop smoking services has moved from Primary Care Trusts to Local Authorities [16–18]. Data from stop smoking services suggests there has been a change in use since 2008 [19] and use of electronic cigarettes has over taken use of prescription medication [20].

There are several reasons why one may expect use of aids for alcohol reduction to be lower than for smoking. First, although the National Treatment Agency for Substance Misuse noted a rise in the number of adults receiving alcohol treatment in 2013–14 [9]; these services are only targeted at those considered to be dependent [21]. In contrast, all smokers, regardless of their cigarette consumption, are advised to attend specialist smoking cessation services if they wish to quit smoking [19]. Secondly, health-care professionals are significantly more likely to discuss an individual’s smoking status, than to offer advice on their alcohol intake [22]. This may be related to differences in financial incentives, confidence in ability to provide advice, and difficulty in the assessment of high-risk drinking [22]. Thirdly, the evidence base for the efficacy of pharmacotherapy and counselling for smoking cessation is stronger having been established across more than a hundred trials [23–29]. In contrast, evidence is limited for the efficacy of licensed medications in maintaining abstinence after detoxification from alcohol [30] and for the therapeutic benefits of drugs (e.g. Baclofen) for alcohol withdrawal [31]. Clinical trials also do not currently support the use of behavioural counselling for high-risk drinking (e.g. motivational interviewing [32]); while the efficacy of self-help groups have not been adequately evaluated [33]. A fourth explanation may be that English stop smoking services are currently more structured and less fragmented than local alcohol services, partially as a consequence of a historically greater focus on tobacco control in public health [34]. Finally, alcohol medications are only available on prescription, while NRT and electronic cigarettes can be purchased in shops and supermarkets. This has likely increased public awareness, along with the advertisement of NRT products in mainstream media since the 1990s [35].

In terms of the characteristics of users of support, the literature suggests that older women are more likely to seek help than younger men for a diverse range of problems such as depression, substance abuse, physical disabilities, and stressful life events [36–41]. Explanations for this include women recognising problems more readily than men, traditional attitudes about the male gender role and heightened feelings of invincibility and propensity for risk-taking during young adulthood [42, 43]. These findings are largely supported by the literature on treatment use for smoking and alcohol [14, 44–46], which also find that socio-economic status (SES) and extent of substance dependency are important. Lower social disadvantage higher cigarette consumption and higher alcohol intake, are associated with greater rates of support [47–51]. Studies on the relationship between ethnicity and drug treatment use suggest that those identifying as being of white ethnicity are more likely to seek help than those of Black and Asian ethnicities [45, 52].

In summary, this study aimed to assess the prevalence of self-reported use of aids to stopping smoking and reducing excessive alcohol consumption in England and establish whether socio-demographic and drug use characteristics differ among those using various aids.

## Methods

### Design

Data were collected on 28,521 participants who took part in the Alcohol Toolkit Study (ATS) and Smoking Toolkit Study (STS) between June 2014 and July 2015. Our included sample was the 25.0% (95%CI 24.5 to 25.5; *n* = 7130) of participants classed as high-risk drinkers (weighted: 26.5%) and 19.1% (95%CI 18.6 to 19.54; *n* = 5426) classed as smokers (weighted: 19.1%). The ATS and STS involve monthly cross-sectional general household computer-assisted interviews, conducted by Ipsos Mori as part of an omnibus survey, of approximately 1,800 adults aged 16+ and over in England. The baseline survey uses a type of random location sampling, which is a hybrid between random probability and simple quota sampling. England is first split into over 170000 ‘Output Areas’, comprising of approximately 300 households. These areas are then stratified according to ACORN socio-demographic characteristics and geographic region (http://www.caci.co.uk/acorn/).

The areas are then randomly allocated to interviewers, who travel to their selected areas starting at a random point and conduct the handheld computer-assisted interviews with one member of the household until interviewers achieve quotas on sex, age, working status and tenure tailored to each area based on census data to minimise differences in the probability of participation. Each monthly survey typically includes ~150 localities. For more details see www.smokinginengland.info and www.alcoholinengland.info or the published protocols [53, 54]. Participants from the STS appear to be representative of the population in England, having similar socio-demographic composition characteristics to large national surveys based on probability samples such as the Health Survey for England [54]. The representativeness of the alcohol-related parameters has yet to be formally assessed, but appears to provide similar estimates as other population surveys

### Inclusion criteria

Our sample consisted of all those aged 16 and over who reported being a smoker and/or met the criteria for a high-risk drinker. Our focus was not on those who were low to moderate risk drinkers, as policy within England focuses on reducing consumption among those who are drinking at hazardous or harmful levels.

### Measures

#### Socio-demographic characteristics

Age, gender, ethnicity and SES were measured. SES was measured using social-grade derived from the National Readership Survey social-grades system [55]: A: higher managerial, administrative or professional; B: intermediate managerial, administrative or professional; C1: supervisory or clerical and junior managerial administrative or professional; C2: skilled manual workers; D: Semi and unskilled manual workers; and E: Casual or lowest grade workers, pensioners and others who depend on the welfare state for their income.

#### Smoking

Participants who reported that they smoked cigarettes daily or non-daily answered the following questions:

1. How many serious attempts to stop smoking have you made in the last 12 months? By serious attempt I mean you decided that you would try to make sure you never smoked again. Please include any attempt that you are currently making and please include any successful attempt made within the last year.
2. Which, if any, of the following did you try to help you stop smoking during your most recent serious quit attempt? [Nicotine replacement therapy on prescription; Nicotine replacement therapy not on prescription; Bupropion; Varenicline; Electronic cigarettes; Attended one or more stop smoking one-to-one counselling\advice\support sessions for help with smoking; Attended a stop smoking group; Phoned a smoking helpline; A self-help book or booklet; Visited a website for smoking cessation; Used an alcohol application ('app') on a handheld computer (smartphone, tablet, PDA); Hypnotherapy; Acupuncture; Nothing/willpower; Don’t know; Other (please specify)]

All participants were asked to complete the Alcohol Use Disorders Identification Test (AUDIT). The AUDIT is used as a method of screening for alcohol use and has validity, high internal consistency and good test-retest reliability across gender, age and cultures [56–58]. The full AUDIT consists of 10 questions: questions 1–3 deal with alcohol consumption (AUDIT-C), 4–6 with alcohol dependence and 7–10 with alcohol-related problems.

Participants scoring 8+ on the full AUDIT [59] or 5+ on questions 1–3 of the AUDIT-C [60, 61], which indicates a hazardous or harmful level of alcohol consumption, answered the following questions:

3. How many attempts to restrict your alcohol consumption have you made in the last 12 months (e.g. by drinking less, choosing lower strength alcohol or using smaller glasses)? Please include all attempts you have made in the last 12 months, whether or not they were successful, AND any attempt that you are currently making.
4. Which, if any, of the following did you use to try to help restrict your alcohol consumption during the most recent attempt? [Any medicines (e.g., acamprosate (Campral), disulfiram (Antabuse), nalmefene (Selincro); Attended one or more one-to-one or group counselling\advice\support sessions for help with drinking; Attended a specialist alcohol clinic or centre for help with drinking; Consulted a community pharmacist for help with drinking; Phoned a helpline for help with drinking (e.g. DrinkLine); An alcohol self-help book or booklet; Visited a website for help with drinking; Used an alcohol application ('app') on a handheld computer (smartphone, tablet, PDA); Hypnotherapy for help with drinking; Acupuncture for help with drinking; Nothing/willpower; Don’t know; Other (please specify)]

### Analysis

All analyses were conducted using R version 3.1.2. Key prevalence figures were calculated both unweighted and weighted to match the population in England. Data were weighted using a rim (marginal) weighting technique. This involves an iterative sequence of weighting adjustments whereby separate nationally representative target profiles are set (for gender, working status, children in the household, age, social-grade and region). This process is then repeated until all variables match the specified targets. For more details see [53]. Linear-by-linear *X* ^2^ tests were used to check for temporal trends in the data that may need to be taken into account in the main analysis. The statistically significant differences in use of the various aids was calculated using Pearson’s *X* ^2^ tests for the association with sex and ethnicity, and using linear-by-linear *X* ^2^ tests for the association with age, social-grade, number of units consumed on a typical day, and cigarette consumption per day. Subsequently, we used generalised linear modelling to regress the use of licensed medication or behavioural support and use of any aid for smoking cessation or alcohol reduction (defined as use of licensed medication, face-to-face counselling, telephone helpline [and NRT over-the-counter for smokers]) on the factors age, sex, social-grade, ethnicity and number of cigarettes/units of alcohol consumed. The quasi-binomial family was specified as there was evidence of overdispersion. As linearity of the logit was violated (assessed using the Box-Tidwell test), age was transformed into a categorical variable.

## Results

Thirty per cent of smokers (95%CI 28.3 to 30.7; *n* = 1600) reported having made at least one quit attempt in the past 12 months; while 12.8% (95%CI 12.0 to 13.6; *n* = 911) of high-risk drinkers reported at least one previous attempt to cut down. Table 1 shows the characteristics of participants who reported a recent quit attempt and/or were high-risk drinkers and had attempted to reduce their intake.

**Table 1:** Number of smokers reporting a recent quit attempt and high-risk drinkers reporting an attempt to cut down stratified by socio-demographic and drug-related characteristics

	Smokers having made a recent quit attempt	High-risk drinkers having made a recent attempt to cut down
Sex %(n)		
*Male*	48.8 (781)	63.3 (577)
*Female*	51.2 (819)	36.7 (334)
Age (years)		
*16–24*	20.9 (335)	14.9 (136)
*25–34*	23.1 (370)	13.5 (123)
*35–44*	17.4 (279)	17.0 (155)
*45–54*	16.2 (260)	22.4 (204)
*55–64*	12.4 (199)	18.7 (170)
*65 and over*	9.8 (157)	13.5 (123)
Social grade		
*AB*	11.1 (177)	34.5 (314)
*C1*	26.6 (425)	34.1 (311)
*C2*	23.5 (376)	13.7 (125)
*D*	19.1 (306)	8.9 (81)
*E*	19.8 (316)	8.8 (80)
Ethnicity %(n)		
*White*	88.3 (1410)	94.7 (859)
*Non–white*	11.7 (186)	5.3 (48)
Cigarette consumption per day %(n)		
*<1*	2.4 (38)	NA
*≥ 1–5*	25.4 (402)	NA
*>5–10*	36.7 (582)	NA
*>10–20*	30.9 (490)	NA
*>20*	4.5 (72)	NA
Units of alcohol on a typical day		
*1–2*	NA	13.2 (120)
*3–4*	NA	37.6 (342)
*5–6*	NA	21.2 (193)
*7–9*	NA	13.1 (199)
*10+*	NA	14.9 (135)
Total %(n)	29.5 (1600)	12.8 (911)

*NA* not applicable

### Overall prevalence of aid use

Linear-by-linear *X*2 analyses did not reveal any evidence for temporal trends in the overall use of aids for attempts to quit smoking or attempts to reduce alcohol intake. There were significant trends for some of the individual aids including NRT use over-the-counter and telephone support for alcohol consumption (see Additional file 1: Table S1). Thus the decision to pool the cross-sectional data was valid and time was not included as a covariate in the analysis of the association between use of aids and socio-demographic and drug-use characteristics.

Table 2 (bottom row) shows that 60.3% of those making a recent quit attempt used an aid to support them, with 31.6% using some form of licensed pharmacological support or formal behavioural support. A significant majority of smokers had used electronic cigarettes (29.1%, *n* = 466). The most commonly used licensed medication was NRT over-the-counter (20.3%, *n* = 325), followed by 5.8% (*n* = 92) using varenicline, 5.1% (*n* = 82) using NRT on prescription and 1.1% (*n* = 17) using bupropion. A small minority of recent quitters used self-help materials and alternative complementary medicine.

**Table 2:** Proportion of smokers using aids during their most recent quit attempt stratified by socio-demographic and smoking characteristics

	Nothing/willpower	Combination of aids							Licensed medication				Electronic cigarettes	Behavioural support			Self-help		Complementary alternative medicine	Other
Strata		Any aid	Licensed medication or behavioural support	Any prescription medication	Any licensed medication	Any behavioural support	Any face-to-face support	Any self-help	NRT on prescription	Varenicline	Buproprion	NRT over-the-counter		Group counselling	Individual counselling	Telephone helpline	Printed	Digital		
Sex %(n)	*					*	*						*							
*Male*	40.5 (316)	58.1 (454)	31.0 (242)	10.8 (84)	30.3 (237)	1.5 (12)	1.5 (12)	1.5 (12)	5.4 (42)	5.0 (39)	0.8 (6)	20.5 (160)	26.8 (209)	0.8 (6)	0.9 (7)	0.0 (0)	0.0 (0)	1.5 (12)	1.3 (10)	2.4 (19)
*Female*	35.3 (289)	62.4 (511)	32.2 (264)	12.2 (100)	31.3 (256)	3.5 (29)	3.5 (29)	1.3 (11)	4.9 (40)	6.5 (53)	1.3 (11)	20.1 (165)	31.4 (257)	1.7 (14)	2.2 (18)	0.0 (0)	0.0 (0)	1.3 (11)	0.7 (6)	2.6 (21)
Age (years)	***	***	***	***	***				**	**	*	**	*		*					
*16–24*	47.2 (158)	50.4 (169)	17.0 (57)	4.2 (14)	16.4 (55)	1.2 (4)	1.2 (4)	2.7 (9)	1.8 (6)	2.4 (8)	0.0 (0)	12.8 (43)	31.3 (105)	0.9 (3)	0.3 (1)	0.0 (0)	0.0 (0)	2.7 (9)	0.3 (1)	2.7 (9)
*25–34*	39.2 (145)	58.1 (215)	27.3 (101)	9.2 (34)	26.2 (97)	1.6 (6)	1.6 (6)	1.1 (4)	5.4 (20)	3.5 (13)	0.5 (2)	17.8 (66)	30.3 (112)	1.1 (4)	0.5 (2)	0.0 (0)	0.0 (0)	1.1 (4)	0.3 (1)	2.2 (8)
*35–44*	31.5 (88)	65.9 (184)	36.9 (103)	14.3 (40)	36.9 (103)	2.9 (8)	2.9 (8)	1.4 (4)	3.9 (11)	10.0 (28)	0.7 (2)	24.4 (68)	33.0 (92)	1.4 (4)	2.2 (6)	0.0 (0)	0.0 (0)	1.4 (4)	0.7 (2)	0.7 (2)
*45–54*	31.5 (82)	67.7 (176)	40.8 (106)	16.9 (44)	39.6 (103)	3.5 (9)	3.5 (9)	1.5 (4)	8.5 (22)	6.9 (18)	2.7 (7)	23.1 (60)	27.7 (72)	1.2 (3)	2.7 (7)	0.0 (0)	0.0 (0)	1.5 (4)	1.9 (5)	3.8 (10)
*55–64*	35.2 (70)	64.3 (128)	38.7 (77)	15.1 (30)	36.7 (73)	5.0 (10)	5.0 (10)	0.5 (1)	8.0 (16)	6.5 (13)	1.0 (2)	23.1 (46)	28.1 (56)	3.0 (6)	2.5 (5)	0.0 (0)	0.0 (0)	0.5 (1)	2.0 (4)	2.5 (5)
*65 and over*	39.5 (62)	59.2 (93)	39.5 (62)	14.0 (22)	39.5 (62)	2.5 (4)	2.5 (4)	0.6 (1)	4.5 (7)	7.6 (12)	2.5 (4)	26.8 (42)	18.5 (29)	0.0 (0)	2.5 (4)	0.0 (0)	0.0 (0)	0.6 (1)	1.9 (3)	3.8 (6)
Social grade																				
*AB*	37.3 (66)	59.9 (106)	34.5 (61)	16.4 (29)	33.9 (60)	2.8 (5)	2.8 (5)	0.6 (1)	8.5 (15)	7.9 (14)	0.6 (1)	18.1 (32)	27.7 (49)	0.6 (1)	2.8 (5)	0.0 (0)	0.0 (0)	0.6 (1)	0.6 (1)	2.3 (4)
*C1*	39.1 (166)	59.8 (254)	31.1 (132)	11.5 (49)	30.6 (130)	1.4 (6)	1.4 (6)	2.4 (10)	3.3 (14)	7.1 (30)	1.4 (6)	19.5 (83)	29.6 (126)	0.5 (2)	1.2 (5)	0.0 (0)	0.0 (0)	2.4 (10)	1.2 (5)	2.6 (11)
*C2*	38.0 (143)	60.4 (227)	29.8 (112)	10.6 (40)	29.0 (109)	2.9 (11)	2.9 (11)	1.1 (4)	5.6 (21)	4.8 (18)	0.8 (3)	19.7 (74)	30.6 (115)	1.3 (5)	1.6 (6)	0.0 (0)	0.0 (0)	1.1 (4)	1.9 (7)	1.3 (5)
*D*	35.9 (110)	61.1 (187)	30.7 (94)	9.8 (30)	29.1 (89)	2.9 (9)	2.9 (9)	1.6 (5)	4.2 (13)	5.6 (17)	0.7 (2)	20.9 (64)	30.1 (92)	2.0 (6)	1.3 (4)	0.0 (0)	0.0 (0)	1.6 (5)	1.0 (3)	2.6 (8)
*E*	38.0 (120)	60.4 (191)	33.9 (107)	11.4 (36)	33.2 (105)	3.2 (10)	3.2 (10)	0.9 (3)	6.0 (19)	4.1 (13)	1.6 (5)	22.8 (72)	26.6 (84)	1.9 (6)	1.6 (5)	0.0 (0)	0.0 (0)	0.9 (3)	0.0 (0)	3.8 (12)
Ethnicity %(n)	**	**											*							
*White*	36.4 (513)	61.8 (871)	32.0 (451)	11.8 (167)	31.2 (440)	2.6 (36)	2.6 (36)	1.6 (22)	5.2 (73)	6.1 (86)	1.1 (15)	20.4 (288)	30.1 (425)	1.3 (19)	1.5 (21)	0.0 (0)	0.0 (0)	1.6 (22)	1.3 (10)	2.4 (19)
*Non-white*	48.4 (90)	49.5 (92)	28.5 (53)	8.6 (16)	27.4 (51)	2.2 (4)	2.2 (4)	0.5 (1)	4.3 (8)	3.2 (6)	1.1 (2)	19.4 (36)	22.0 (41)	0.5 (1)	1.6 (3)	0.0 (0)	0.0 (0)	0.5 (1)	0.7 (6)	2.6 (21)
Cigarette consumption per day %(n)	***	***	***	***	***					***			*							
*<1*	55.3 (21)	44.7 (17)	21.1 (8)	5.3 (2)	21.1 (8)	0.0 (0)	0.0 (0)	2.6 (1)	2.6 (1)	2.6 (1)	0.0 (0)	15.8 (6)	18.4 (7)	0.0 (0)	0.0 (0)	0.0 (0)	0.0 (0)	2.6 (1)	0.0 (0)	2.6 (1)
*≥ 1–5*	46.0 (185)	51.7 (208)	23.1 (93)	6.7 (27)	22.6 (91)	2.0 (8)	2.0 (8)	2.2 (9)	4.2 (17)	2.2 (9)	0.5 (2)	17.2 (69)	26.4 (106)	0.5 (2)	1.7 (7)	0.0 (0)	0.0 (0)	2.2 (9)	0.7 (3)	2.7 (11)
*>5–10*	40.0 (233)	57.9 (337)	30.8 (179)	10.3 (60)	29.9 (174)	2.2 (13)	2.2 (13)	0.9 (5)	4.6 (27)	5.2 (30)	0.7 (4)	19.9 (116)	28.2 (164)	0.9 (5)	1.5 (9)	0.0 (0)	0.0 (0)	0.9 (5)	0.5 (3)	2.1 (12)
*>10–20*	30.2 (148)	68.4 (335)	39.6 (194)	16.7 (82)	38.6 (189)	3.7 (18)	3.7 (18)	1.4 (7)	6.5 (32)	9.2 (45)	1.8 (9)	23.3 (114)	30.6 (150)	2.2 (11)	1.8 (9)	0.0 (0)	0.0 (0)	1.4 (7)	1.6 (8)	2.4 (12)
*>20*	18.1 (13)	79.2 (57)	38.9 (28)	16.7 (12)	37.5 (27)	2.8 (2)	2.8 (2)	1.4 (1)	5.6 (4)	9.7 (7)	2.8 (2)	22.2 (16)	44.4 (150)	2.8 (2)	0.0 (0	0.0 (0)	0.0 (0)	1.4 (1)	2.8 (2)	4.2 (3)
Total %(n)	37.8 (605)	60.3 (965)	31.6 (506)	11.5 (184)	30.8 (493)	2.6 (41)	2.6 (41)	1.4 (23)	5.1 (82)	5.8 (92)	1.1 (17)	20.3 (325)	29.1 (466)	1.3 (20)	1.6 (25)	0.0 (0)	0.0 (0)	1.4 (23)	1.0 (16)	2.5 (40)

Figures are presented as % (n) within stratum; those who reported that they used hypnosis or acupuncture were classed as having used an alternative complementary medicine; those using a website or mobile phone application were grouped as having used an digital intervention; any aid includes all aids listed; any prescription medication includes NRT on prescription, varenilcine and bupropion; any licensed medication includes any prescription medication and NRT over-the-counter; any behavioural support includes any face-to-face support and telephone helpline; any face-to-face support includes group counselling, individual counselling and telephone helpline; any self-help includes printed and digital interventions; *P* < 0.05, **P < 0.01, ***P < 0.001 (by linear-by-linear *X*2 tests or Pearson’s *X*2 test)

Table 3 (bottom row) shows that a substantially smaller number of those cutting down on alcohol used an aid (14.9%, *n* = 136) relative to those attempting to quit smoking, with just 5.7% using medically licensed pharmacotherapy or behavioural support. Use of self-help materials was more common than for quitting smoking (4.1%, n= 38), but fewer used complementary alternative medicines (0.5%, *n* = 5).

**Table 3:** Proportion of drinkers using aids during their most recent attempt to cut down stratified by socio-demographic and drinking characteristics

		Combination of aids						Behavioural support				Self-help			
Strata	Nothing/Willpower	Any aid	Licensed medication or behavioural	Licensed medication	Any behavioural support	Any face-to-face support	Any self-help	Group counselling	Specialist clinic	Community pharmacist	Telephone helpline	Printed	Digital	Complementary alternative medicine	Other
Sex %(n)									*						*
*Male*	79.0 (456)	13.7 (79)	5.9 (34)	0.9 (5)	5.7 (33)	5.5 (32)	4.0 (23)	2.4 (14)	3.8 (22)	0.5 (3)	0.7 (4)	1.6 (9)	2.9 (17)	0.5 (3)	5.5 (32)
*Female*	76.6 (256)	17.1 (57)	5.4 (18)	1.8 (6)	3.9 (13)	3.6 (12)	3.3 (11)	2.4 (8)	0.9 (3)	0.6 (2)	0.6 (2)	1.5 (5)	2.1 (7)	0.6 (2)	9.0 (30)
Age (years)						*									
*16–24*	82.4 (112)	11.8 (16)	7.4 (10)	0.7 (1)	6.6 (9)	6.6 (9)	4.4 (6)	1.5 (2)	3.7 (5)	1.5 (2)	0.7 (1)	2.9 (4)	2.9 (4)	0.0 (0)	3.7 (5)
*25–34*	80.5 (99)	14.6 (18)	5.7 (7)	0.8 (1)	4.9 (6)	4.9 (6)	4.1 (5)	2.4 (3)	2.4 (3)	0.0 (0)	0.0 (0)	0.8 (1)	4.1 (5)	0.8 (1)	5.7 (7)
*35–44*	74.2 (115)	20.6 (32)	9.7 (15)	2.6 (4)	7.7 (12)	7.7 (12)	4.5 (7)	3.9 (6)	4.5 (7)	0.6 (1)	0.6 (1)	1.9 (3)	2.6 (4)	0.0 (0)	8.4 (13)
*45–54*	79.4 (162)	14.7 (30)	5.4 (11)	1.5 (3)	5.4 (11)	4.9 (10)	4.9 (10)	3.4 (7)	2.9 (6)	1.0 (2)	1.5 (3)	2.0 (4)	2.9 (6)	1.5 (3)	5.9 (12)
*55–64*	78.2 (113)	13.5 (23)	3.5 (6)	0.6 (1)	3.5 (6)	3.5 (6)	2.4 (4)	1.8 (3)	2.4 (4)	0.0 (0)	0.0 (0)	0.6 (1)	2.4 (4)	0.0 (0)	8.2 (14)
*65 and over*	74.0 (91)	13.8 (17)	2.4 (3)	0.8 (1)	1.6 (2)	0.8 (1)	1.6 (2)	0.8 (1)	0.0 (0)	0.0 (0)	0.8 (1)	0.8 (1)	0.8 (1)	0.8 (1)	8.9 (11)
Social grade	***	***	***	***	***	***		**	***		**	**			
*AB*	80.6 (253)	12.1 (38)	1.9 (6)	0.3 (1)	1.9 (6)	1.9 (6)	2.5 (8)	1.6 (5)	1.0 (3)	0.3 (1)	0.3 (1)	0.6 (2)	2.2 (7)	0.0 (0)	8.0 (25)
*C1*	81.4 (253)	12.5 (39)	5.1 (16)	1.3 (4)	4.2 (13)	4.2 (13)	3.2 (10)	1.9 (6)	1.9 (6)	0.6 (2)	0.0 (0)	1.3 (4)	2.3 (7)	0.6 (2)	5.5 (17)
*C2*	79.3 (99)	12.8 (16)	4.0 (5)	0.0 (0)	4.0 (5)	4.0 (5)	3.2 (4)	0.8 (1)	2.4 (3)	0.8 (1)	0.8 (1)	1.6 (2)	2.4 (3)	1.6 (2)	5.6 (7)
*D*	72.8 (59)	23.5 (19)	13.6 (11)	0.0 (0)	13.6 (11)	11.1 (9)	6.2 (5)	4.9 (4)	6.2 (5)	1.2 (1)	2.5 (2)	2.7 (3)	3.7 (3)	1.2 (1)	7.4 (6)
*E*	60.0 (48)	30.0 (24)	17.5 (14)	7.5 (6)	13.8 (11)	13.8 (11)	8.8 (7)	7.5 (6)	10.0 (8)	0.0 (0)	2.5 (2)	3.8 (3)	5.0 (4)	0.0 (0)	8.8 (7)
Ethnicity %(n)	*	**	**		***	**	**	*		*			**		
*White*	78.9 (678)	14.1 (121)	5.0 (43)	1.2 (10)	4.3 (37)	4.2 (36)	3.3 (28)	2.0 (17)	2.8 (24)	0.3 (3)	0.5 (4)	1.4 (12)	2.2 (19)	0.6 (5)	6.9 (59)
*Non-white*	64.6 (31)	29.2 (14)	16.7 (8)	2.1 (1)	16.7 (8)	14.6 (7)	12.5 (6)	8.3 (4)	2.1 (1)	4.2 (2)	4.2 (2)	4.2 (2)	10.4 (5)	0.0 (0)	6.2 (3)
Units of alcohol on a typical day									**						
*1–2*	77.9 (95)	14.8 (18)	5.8 (7)	2.5 (3)	5.0 (6)	6.6 (8)	1.7 (2)	2.5 (3)	4.2 (5)	0.0 (0)	0.8 (1)	0.8 (1)	1.6 (2)	1.6 (2)	5.7 (7)
*3–4*	76.9 (263)	15.2 (52)	4.7 (16)	0.3 (1)	4.7 (16)	4.7 (16)	3.8 (13)	2.9 (10)	1.5 (5)	0,3 (1)	0.3 (1)	1.5 (5)	2.6 (9)	0.3 (1)	8.2 (28)
*5–6*	76.7 (148)	16.6 (32)	4.1 (8)	1.0 (2)	3.1 (6)	2.6 (5)	5.7 (11)	1.0 (2)	1.0 (2)	0.5 (1)	0.5 (1)	3.1 (6)	3.6 (7)	0.0 (0)	8.3 (16)
*7–9*	82.4 (98)	12.6 (15)	5.0 (6)	0.8 (1)	4.2 (5)	3.4 (4)	3.4 (4)	0.0 (0)	1.7 (2)	1.7 (2)	0.8 (1)	0.8 (1)	3.4 (4)	0.8 (1)	4.2 (5)
*10+*	108 (80)	14.1 (19)	9.6 (13)	3.0 (4)	8.1 (11)	8.1 (11)	2.2 (3)	3.7 (5)	6.7 (9)	0.7 (1)	1.5 (2)	0.7 (1)	1.5 (2)	0.7 (1)	4.4 (6)
Total %(n)	78.2 (712)	14.9 (136)	5.7 (52)	1.2 (11)	5.0 (46)	4.8 (44)	3.7 (34)	2.4 (22)	2.7 (25)	0.5 (5)	0.7 (6)	1.5 (14)	2.6 (24)	0.5 (5)	6.8 (62)

Figures are presented as % (n) within stratum; those who reported that they used hypnosis or acupuncture were classed as having used an alternative complementary medicine; those using a website or mobile phone application were grouped as having used an digital intervention; any aid includes all aids listed; any behavioural support includes any face-to-face support and telephone helpline; any face-to-face support includes group counselling, specialist clinic and community pharmacist; any self-help includes printed and digital interventions; *P* < 0.05, **P < 0.01, ***P < 0.001 (by linear-by-linear *X*2 tests or Pearson’s *X*2 test)

### Associations between aid use and socio-demographic and smoking characteristics

A higher proportion of female (3.5%) than male (1.5%) smokers who had tried to quit smoking in the past 12 months had used behavioural support (Table 2); while a higher proportion of recent male quitters (40.5%) reported using willpower alone than recent female quitters (35.3%). There was an increase in the use of some form of smoking cessation aid generally (and medically licensed pharmacotherapy or behavioural support) with age, which appeared to stem from the greater use of medication among older age groups. Those of white ethnicity were more likely to use support, with the difference resulting mainly from a greater use of electronic cigarettes. Electronic cigarette users also tended to be younger. In contrast, those of non-white ethnicity were more likely to report use of will-power alone. Use of aids generally (and medically licensed pharmacotherapy or behavioural support) appeared to be more common among those smoking a greater number of cigarettes per day, again this appeared to stem from a greater use of licensed medication. There were no differences as a function of social-grade.

A greater proportion of female (9.0%) than male (5.5%) drinkers who had attempted to cut down on their alcohol had used a different aid than those listed (Table 3). Use of face-to-face support decreased with increasing age, while use of most aids was more common among lower social-grades. For example, 17.5% of those classed as social-grade E used medically licensed pharmacotherapy and behavioural support compared with just 1.9% of those in social-grade AB. In relation to ethnicity, those of non-white ethnicity were more likely to use an aid than those of white ethnicity (29.2% versus 14.1%). This appeared to stem from a greater use of behavioural support and self-help. There were no differences as a function of the number of units consumed on a typical day expect among those using a specialist clinic, with the prevalence of use highest among those consuming more than 10 units.

Table 4 shows the multiple logistic regression models in which each factor associated with use of aids and licensed medication or behavioural support were adjusted for other factors. The odds of using aids, licensed medication or behavioural support for smoking cessation was higher among all age groups relative to those aged 16 to 24 (expect those aged 65+). The odds of using aids and licensed medication or behavioural support for smoking cessation were also higher among those smoking more than 10 cigarettes per day relative to those smoking less than 1; while use of aids generally was lower among ethnic minority groups. In contrast, the use of aids and licensed medication or behavioural support to cut down on drinking was higher among those of social grade D and E and those of non-white ethnicity.

**Table 4:** Multiple logistic regression model regarding the use of aids and licensed medication and/or behavioural specifically (yes versus no) on socio-demographic and behaviour-related factors in smokers making a recent quit attempt and drinkers attempting to cut down

	Use of aids among smokers attempting to quit (*n* = 965 versus *n* = 635)				Use of aids among drinkers attempting to cut down (*n* = 136 versus *n* = 775)				Use of licensed medication or behavioural among smokers attempting to quit (*n* = 506 versus *n* = 1094)				Use of licensed medication or behavioural among drinkers attempting to cut down (*n* = 52 versus *n* = 859)			
		95%CI				95%CI				95%CI				95%CI		
	OR	Lower	Upper	*P*	OR	Lower	Upper	*P*	OR	Lower	Upper	*P*	OR	Lower	Upper	*P*
Sex %(n)																
*Male*	1				1				1				1			
*Female*	1.14	0.93	1.43	0.192	1.24	0.84	1.85	0.278	1.04	0.83	1.30	0.748	0.89	0.44	1.73	0.731
Age (years)																
*16–24*	1				1				1				1			
*25–34*	1.49	1.09	2.03	**0.012**	1.31	0.61	2.83	0.491	1.99	1.37	2.92	**<0.001**	0.81	0.24	2.47	0.716
*35–44*	1.93	1.38	2.72	**<0.001**	1.98	1.01	4.01	0.050	2.94	2.00	4.36	**<0.001**	1.63	0.64	4.27	0.308
*45–54*	1.93	1.36	2.74	**<0.001**	1.43	0.72	2.90	0.313	3.31	2.25	4.93	**<0.001**	1.00	0.36	2.76	0.992
*55–64*	1.66	1.14	2.42	**0.008**	1.36	0.66	2.85	0.412	3.10	2.05	4.72	**<0.001**	0.77	0.23	2.39	0.661
*65 and over*	1.35	0.91	2.01	0.135	1.47	0.66	3.26	0.345	3.19	2.06	4.98	**<0.001**	0.52	0.10	2.03	0.379
Social grade																
*AB*	1				1	1	1		1				1			
*C1*	1.13	0.77	1.64	0.534	1.08	0.65	1.78	0.766	1.09	0.74	1.62	0.656	2.30	0.86	6.99	0.112
*C2*	1.03	0.70	1.50	0.882	1.06	0.54	1.99	0.860	0.88	0.59	1.31	0.530	1.81	0.47	6.58	0.365
*D*	1.06	0.71	1.58	0.762	2.29	1.19	4.34	**0.012**	1.02	0.67	1.55	0.928	6.57	2.24	21.17	**0.001**
*E*	0.99	0.66	1.47	0.951	3.13	1.69	5.73	**<0.001**	1.12	0.74	1.69	0.598	8.63	3.02	27.40	**<0.001**
Ethnicity																
%(n)	1				1		1		1	1			1			
*White* *Non-white*	0.69	0.50	0.96	**0.026**	2.51	1.23	4.90	**0.009**	1.00	0.69	1.43	0.996	4.01	1.51	9.68	**0.003**
Cigarette																
consumption	1								1	1			*-*	*-*	*-*	*-*
per day %(n)	1.30	0.64	2.62	0.466	*-*	*-*	*-*	*-*	1.23	0.56	3.01	0.631	*-*	*-*	*-*	*-*
*<1*	1.66	0.84	3.34	0.147	*-*	*-*	*-*	*-*	1.82	0.84	4.41	0.153	*-*	*-*	*-*	*-*
*≥1-5*	2.47	1.23	5.00	**0.011**	*-*	*-*	*-*	*-*	2.50	1.15	6.08	**0.029**	*-*	*-*	*-*	*-*
*>5-10*	4.23	1.77	10.47	**0.001**	*-*	*-*	*-*	*-*	2.26	0.91	6.06	0.089	*-*	*-*	*-*	*-*
*>10-20* *>20*																
Units of	*-*	*-*	*-*	*-*												
alcohol on a	*-*	*-*	*-*	*-*	1				*-*	*-*	*-*	*-*	1			
typical day	*-*	*-*	*-*	*-*	1.16	0.64	2.18	0642	*-*	*-*	*-*	*-*	0.91	0.34	2.78	0.856
*1–2*	*-*	*-*	*-*	*-*	1.20	0.63	2.36	0.593	*-*	*-*	*–*	*-*	0.62	0.19	2.09	0.420
*3–4*	*-*	*-*	*-*	*-*	0.94	0.43	2.04	0.875	*-*	*-*	*-*	*-*	0.85	0.24	3.07	0.804
*5–6*	*-*	*-*	*-*	*-*	1.04	0.48	2.23	0.928	*-*	*-*	*-*	*-*	1.46	0.248	4.87	0.519
*7–9* *10+*																

Note: licensed medication and/or behavioural for smoking cessation includes prescription medication, NRT over-the-counter, face-to-face counselling and telephone helpline; licensed medication and/or behavioural for alcohol includes licensed medication, face-to-face support and telephone helpline

## Discussion

A majority of smokers trying to stop used some form of aid, whereas this was true for only a small minority of people trying to reduce their alcohol consumption. Pharmacological support was by far the most commonly used aid for smokers but was used very little for alcohol reduction. Use of face-to-face counselling was low in both groups but slightly higher for people reducing their alcohol consumption than smokers trying to stop. The use of support was greater among older smokers with higher cigarette consumption, and lower among those of non-white ethnicity. In contrast, use of aids during attempts to reduce alcohol intake was more common among ethnic minority groups and those of social-grades D and E relative to AB.

The finding that only 1/3rd of smokers in England were using medically licensed pharmacotherapy or behavioural support is significantly lower than the 51.2% in 2008 [14], but is close to the estimated 36.1% of smokers using treatment in the US [46]. This appears to reflect a decline in use of behavioural support, use of prescription medication and use of NRT over-the-counter; findings which are in line with audit data from the English Stop Smoking Services [19]. Sales of NRT have been decreasing steadily in recent years [20], a trend which does not appear to be a consequence of the rise in electronic cigarettes but may reflect a longer term disengagement from licensed nicotine products [62]. The reduction in use of behavioural support may be attributable to the move of commissioning of stop smoking services from Primary Care Trusts to Local Authorities, which has created a fragmented system of support [63].

In line with surveys from the US, Europe, and Brazil [47, 64, 65], only a small proportion of participants used aids during attempts to reduce their alcohol intake. This could be due to lack of availability and/or awareness [66, 67]. For example, many drinkers are unaware that they are consuming alcohol at harmful levels, and few receive advice from healthcare professionals to counteract this [22, 68]. In contrast, most smokers acknowledge that cigarettes are harmful to their health [69]. Thus there is a need for increasing intervention rates among high risk drinkers [70, 71]. These discrepancies may stem from a social and cultural acceptance of drinking in contrast to the stigmatisation of smoking [72]; alcohol marketing and promotion [73], or some of the adverse consequences of alcohol consumption not being evaluated in a negative manner [74].

The majority of the difference in use of aids among smokers and high-risk drinkers was attributed to use of licensed medication. The less prevalent use among drinkers likely results from availability, with medications to prevent alcohol withdrawal symptoms only available on prescription and targeted at the most dependent drinkers. In contrast, smoking pharmacotherapy is available on prescription and over-the-counter [27, 28, 35, 66, 67]. Use of electronic cigarettes was also high among this sample. Studies suggest that electronic cigarettes may be a suitable substitute for traditional tobacco containing products [24, 25, 75]. In contrast, the finding that use of face-to-face support among smokers was lower than that for alcohol reduction is surprising given that England has a national network of stop-smoking clinics that are quite heavily promoted. This is of concern since both group and individual behavioural support appear to be efficacious in promoting quit attempts and ensuring the success of those attempts [27]. Thus, it will be important to find out how its use can be increased. A significantly greater minority of high-risk drinkers were also using self-help materials. Policy makers should take note of this as it may reflect a strong preference for aids which do not require one to engage in face-to-face behavioural support [76].

It is perhaps unsurprising that smokers with higher cigarette consumption had greater odds of reporting use of licensed medication and/or behavioural support and aids generally. More dependent smokers may be more inclined to seek help as a consequence of continued failures to stop and the experience of stronger withdrawal symptoms [76]. In contrast, despite specialist alcohol services being aimed at more dependent drinkers, no association was found with the average number of units consumed per day. This may reflect the failure of GPs to identify and refer the most dependent drinkers [22], the fact that alcohol dependency is multifaceted and cannot be measured accurately with a single item [58], or the cross-sectional nature of study, whereby any reduction is precluded by the fact that those seeking treatment were of higher dependency initially. The finding that those of older age were more likely to seek help is consistent with previous studies, although the effect of gender was much weaker [14, 36, 42]. The lack of association between licensed medication and/or behavioural support for smoking cessation and social-grade may reflect the fact that those from lower social-grades do not need to pay for pharmacotherapy prescriptions. In contrast, high-risk drinkers of lower SES and of non-white ethnicity were more likely to seek help. This could be due to cultural norms regarding alcohol use, with greater stigmatisation among minority ethnic groups leading to a higher motivation to seek help. Research is also accumulating to suggest that those of lower social-grades experience greater harm from their alcohol use and report higher dependency [77].

The study had several limitations. First, there is a risk that respondents will underestimate or fail to report their drinking and smoking and use of aids. Secondly, the findings are limited to a single country, albeit one from which useful lessons may be learned internationally given its unique approach to treatment provision. Thirdly, there are intrinsic limitations to population surveys that warrant caution in extrapolating to populations including the under-representation of students, the homeless and other vulnerable populations. Fourthly, this study was limited to investigating the overall prevalence of use of aids by smokers and high-risk drinkers. Given the strong association between tobacco and alcohol use [78], research into the use of aids to quitting smoking and reducing excessive alcohol consumption by smokers consuming excessive alcohol use could also be informative. Finally, conclusions regarding the effectiveness of various aids cannot be made due to the cross-sectional design and the exclusion of those who have successfully quit smoking or reduced their alcohol consumption to low/moderate levels. This study does provide an indication of the current prevalence of use of aids and the characteristics of those most likely to opt for help during attempts to quit or cut down on their smoking and alcohol intake.

## Conclusion

In conclusion, this study finds that a majority of smokers are using some form of aid to smoking cessation and that this primarily involves pharmacological support. Use of face-to-face behavioural support, while free, remains low and higher for alcohol reduction than smoking cessation. There are important sociodemographic differences in the use of aids to smoking cessation and alcohol use reduction.

## Additional file

Additional file 1: Table S1. Trend in aid use among smokers and high-risk drinkers over the study period. (DOC 54 kb)

## Abbreviations

STS: Smoking toolkit study
ATS: Alcohol toolkit study
NRT: Nicotine replacement therapy
