# Supplementary material for: Use of aids for smoking cessation and alcohol reduction: A population survey of adults in England
Source: BMC Public Health. 2016 Dec 8;16:1237. doi: 10.1186/s12889-016-3862-7 (PMC5146832; doi:10.1186/s12889-016-3862-7)
Supplement: Additional file 1: Table S1. — Trend in aid use among smokers and high-risk drinkers over the study period. (DOC 54 kb) [file 12889_2016_3862_MOESM1_ESM.doc]

Additional file 1: Table S1. Trend in aid use among smokers and high-risk drinkers over the study period

|  | Smokers  X^2^-value (p) | High-risk drinkers  X^2^-value (p) |
| --- | --- | --- |
| Any aid | 0.31 (0.577) | 2.86 (0.090) |
| Prescription medication | <0.01 (0.969) | 0.08 (0.775) |
| NRT on prescription | 0.45 (0.501) | NA |
| NRT over-the-counter | 9.35 (0.002) | NA |
| Varenicline | 0.30 (0.586) | NA |
| Bupropion | 1.29 (0.256) | NA |
| Electronic cigarettes | 2.04 (0.153) | NA |
| Counselling | 0.27 (0.605) | 2.58 (0.109) |
| Digital interventions | 1.04 (0.307) | 1.41 (0.235) |
| Self-help books | NA^a^ | 2.58 (0.109) |
| Telephone support | NA^a^ | 3.98 (0.046) |
| Complementary alternative medicine | 0.14 (0.709) | 0.36 (0.551) |
| Other aid | 5.99 (0.014) | 1.14 (0.286) |

By linear-by-linear X^2^ tests; NA= not applicable; ^a^ zero prevalence over the study period
